# Genetic blueprint of congenital muscular dystrophies with brain malformations in Egypt: A report of 11 families

**DOI:** 10.1007/s10048-024-00745-z

**Published:** 2024-02-01

**Authors:** Sylvia Safwat, Kyle P. Flannery, Ahmed A. El Beheiry, Mohamed M. Mokhtar, Ebtesam Abdalla, M. Chiara Manzini

**Affiliations:** 1 Department of Neuroscience and Cell Biology, Child Health Institute of New Jersey, Rutgers-Robert Wood Johnson Medical School, New Brunswick, NJ USA; 2 Department of Human Genetics, Medical Research Institute, Alexandria University, Alexandria, Egypt; 3 Department of Radiodiagnosis and Interventional Radiology, Faculty of Medicine, Alexandria University, Alexandria, Egypt

**Keywords:** Congenital muscular dystrophy, Lissencephaly, Hydrocephalus, Dystroglycanopathy, FLVCR1

## Abstract

Congenital muscular dystrophies (CMDs) are a group of rare muscle disorders characterized by early onset hypotonia and motor developmental delay associated with brain malformations with or without eye anomalies in the most severe cases. In this study, we aimed to uncover the genetic basis of severe CMD in Egypt and to determine the efficacy of whole exome sequencing (WES)-based genetic diagnosis in this population. We recruited twelve individuals from eleven families with a clinical diagnosis of CMD with brain malformations that fell into two groups: seven patients with suspected dystroglycanopathy and five patients with suspected merosin-deficient CMD. WES was analyzed by variant filtering using multiple approaches including splicing and copy number variant (CNV) analysis. We identified likely pathogenic variants in *FKRP* in two cases and variants in *POMT1*, *POMK*, and *B3GALNT2* in three individuals. All individuals with merosin-deficient CMD had truncating variants in *LAMA2*. Further analysis in one of the two unsolved cases showed a homozygous protein-truncating variant in *Feline Leukemia Virus subgroup C Receptor 1* (*FLVCR1*). *FLVCR1* loss of function has never been previously reported. Yet, loss of function of its paralog, *FLVCR2*, causes lethal hydranencephaly-hydrocephaly syndrome (Fowler Syndrome) which should be considered in the differential diagnosis for dystroglycanopathy. Overall, we reached a diagnostic rate of 86% (6/7) for dystroglycanopathies and 100% (5/5) for merosinopathy. In conclusion, our results provide further evidence that WES is an important diagnostic method in CMD in developing countries to improve the diagnostic rate, management plan, and genetic counseling for these disorders.

## Introduction

Congenital muscular dystrophies (CMDs) are a clinically and genetically heterogeneous group of disorders characterized by hypotonia within the first year of life, motor development delay, and progressive muscle weakness in addition to dystrophic features on muscle biopsy and elevated creatine kinase (CK) [1]. Brain malformations, with or without accompanying eye anomalies, are key features of severe forms of CMD such as the dystroglycanopathies [including Muscle-Eye-Brain (MEB), Fukuyama CMD (FCMD) and Walker-Warburg syndrome (WWS)] and merosinopathies (merosin-deficient CMD).

To date, mutations in 18 genes have been reported to cause dystroglycanopathies, each of which are involved in glycosylation of α-dystroglycan (α- DG) [2], while merosin-deficient CMD is caused by mutations in the laminin-α2 gene (*LAMA2*), a component of the laminin subtype, merosin [3]. Together, these genes functionally converge on the regulation of interactions between cells and the basement membrane and extracellular matrix (ECM), as the functional glycan on α-DG binds ECM ligands such as merosin and other laminin G-like domain-containing proteins [4].

The worldwide prevalence of all CMDs ranges from 0.5 to 0.9 per 100,000 [5, 6]. In Egypt, one study in 2005 reported that the prevalence of combined muscular dystrophies was 26.8 per 100,000, while CMD prevalence was as high as 3.8 per 100,000 [7]. The prevalence of more severe forms of dystroglycanopathy with brain and eye malformations (MEB, FCMD, and WWS) are more difficult to quantify due to their early lethality. Studies focused on geographical distribution of mutations in severe dystroglycanopathy have been helpful in defining the most common genetic causes. European cohorts have identified mutations in *POMT1* as the most frequent cause of WWS [5, 8], but this also held true in an ethnically diverse cohort [8]. In contrast, *POMGNT1* mutations are the most frequent cause of MEB overall [9]. However, a founder retrotransposon insertion in the 3’ UTR of *FKTN* contributes to the majority of CMDs in Japan [10], and this same insertion was also identified in 14/42 dystroglycanopathy patients in a South Korean cohort [11]. Similarly, a different founder mutation in *FKTN* with a carrier frequency of 0.7% leads to WWS in the Ashkenazi Jewish population [12]. Merosinopathy is the most common CMD in the United Kingdom [5], the 2nd and the 3rd most common cause in Italy and Australia respectively [6, 13]. However, it accounts for only 6% of CMD in Japan [14]. As such, regional differences can be expected.

Next-generation sequencing (NGS) technologies such as whole-exome sequencing (WES) or gene panels have had a great impact on the medical diagnosis of CMDs, increasing the speed of genetic diagnosis in developed countries [15] and even in developing countries according to recent studies in Africa and Jordan [16, 17]. As multiple drug and gene therapy-based approaches are being approved or tested for other forms of muscular dystrophy, it is imperative to define the genetic distribution of CMD mutations in different populations which could benefit from future therapies.

Despite global progress, CMDs remain understudied in developing countries. To address this gap, we recruited 12 patients from 11 Egyptian families presenting with CMDs with brain involvement. The overarching goal of this study is to provide a genetic diagnosis for each patient, which can improve traditional methods of diagnosis and genetic counseling, as well as treatment and research strategies.

## Materials and methods

### Subjects

Ethics approval was received from the Ethics Committee of Medical Research Institute and Faculty of Medicine, Alexandria University and the Institutional Review Board at Rutgers University. All patients/guardians were fully informed of the purpose and procedures of this study and written consent was obtained before participation. A full clinical assessment, pedigree, and family history for all patients was completed at the Human Genetic Clinic, Medical Research Institute in Alexandria, Egypt.

### Whole exome sequencing and analysis

Genomic DNA was extracted from blood samples using a QIAmp DNA Mini Kit (Qiagen). Library preparation and whole exome sequencing was performed by Novogene Corporation Inc. (Sacramento, CA) using a SureSelect Human All Exon V6 kit (Agilent) and NovaSeq 6000 sequencer. Sequencing reads were mapped to hg38 with BWA [18], sorted with Sambamba [19], and merged with Picard, and variants were called with GATK [20], as part of Novogene’s Bioinformatics Analysis pipeline. Variant call format (VCF) files were then annotated with ANNOVAR [21], stored in a custom SQL database, and filtered for variants with allele frequencies < 1% in gnomAD v3.1.2 and the Greater Middle Eastern Variome (GME) Northeast Africa and Arabian Peninsula populations [22]. Pathogenicity predictors including SIFT [23], PolyPhen2 [24], and CADD [25] were used to assess the effect of missense variants, and SpliceAI [26] was used to evaluate intronic and synonymous variants for their potential to disrupt splicing. In addition, ExomeDepth [27] was used to identify any copy number variants (CNVs) affecting genes known to cause CMD using unrelated individuals as controls.

## Results

### Clinical description

All individuals in our cohort presented with early onset hypotonia, brain abnormalities in magnetic resonance imaging (MRI), myopathic changes on EMG, and/or elevated serum creatinine kinase suggestive of CMD. However, the overall clinical and radiological presentation defined 2 groups. Group I included seven patients (four females and three males) with suspected dystroglycanopathy, and Group II was comprised of five patients (one female and four males) with suspected merosin-deficient CMD (Table 1). Clinical assessment alone was not enough for a definitive diagnosis in S7, however, as he exhibited manifestations of both MEB and merosin-deficient CMD.

**Table 1:** Summary of clinical findings

	Suspected dystroglycanopathy							Suspected merosin-deficient CMD (MDC1A)				
	S1	S2	S3	S4	S5	S6	S7	S8	S9	S10	S11	S12
Sex	F	M	F	M	F	F	M	F	M	M	M	M
Consanguinity	1^st^ cous	No	1^st^ cous	1^st^ cous OR	1^st^ cous	1^st^ cous	1^st^ cous	2^nd^ cous	1^st^ cous	2^nd^ cous	1^st^ cous	1^st^ cous
Age at 1^st^ visit	1y 6mo	15 days	1 day	7 days	1mo	7mo	1y 3m	2y	2y 10 mo	7y	4y 6mo	3y 4mo
Age	5y	3y 6mo	Died (8mo)	Died (11mo)	Died (6mo)	Died (2y)	2y 10mo	5y 3mo	6y	9y	5y 8mo	4y 6mo
Head control	-	-	-	-	-	-	Delay	Delay	Delay	Delay	Delay	Delay
Sitting	-	-		-	-	-	Delay	Delay	Delay	-	+	+
Walking	-	-				-	-	-	-	-	-	-
Speech	-	-				-	-	+	Delay	+	Delay	Delay
Contractures	-	-	-	-	+	-	-	+	-	+	+	-
CPK	5,000	3,283	High	3,534	2,030	n/a	13–330	418	1,601	175	173	561
Myopathy upon EMG	+	n/a	+	n/a	n/a	+	+	+	+	+	+	+
Hydrocephalus	+ (shunt)	-	+ (shunt)	+	-	+ (shunt)	-	-	-	-	-	-
Cortical malformation	Cobblestone	Dysplasia		Cobblestone	Lissencephaly	Cobblestone	ND	Occipital pachygyria	ND	ND	ND	ND
Cerebellar malformation	Hypoplastic vermis, brain stem kink	Hypoplastic, microcysts, small pons with ventral notch	Microcysts	Hypoplastic, brain stem kink	Hypoplastic/ dysplastic, brain stem kink	Hypoplastic, microcysts, small pons with ventral notch	Hypoplasia, microcysts	ND	Microcysts	ND	ND	ND
White matter hyperintensity	-	-	-	-	-	-	+	+	+	+	+	+
Seizures	+	-	+	+	+	+	-	-	-	-	-	-
Eye malformation	Bilateral retinal dysplasia	R severe MO, microcornea, PHPV	Corneal opacity	L severe MO, soft IOP R optic disc hypoplasia	Bilateral atrophic hypoplastic ON head	Nystagmus	L mild ptosis	ND	ND	ND	ND	ND
Diagnosis	WWS	MEB	WWS	WWS	WWS	WWS	MEB? MDC1A?	MDC1A	MDC1A	MDC1A	MDC1A	MDC1A

+ or – indicates the presence or absence of a sign. Some cells are left blank because the patient died before milestone could be reached. Abbreviations: F, female; M, male; cous, cousin; OR, once removed; y, year; mo, month; R, right; L, left; MO microphthalmia; PHPV, persistent hyperplastic primary vitreous; IOP, intra-ocular pressure; ON, optic nerve; n/a, not available; ND, not detected; WWS, Walker Warburg Syndrome; MEB, Muscle Eye Brain disease; MDC1A, merosin-deficient CMD

**Fig. 1:**
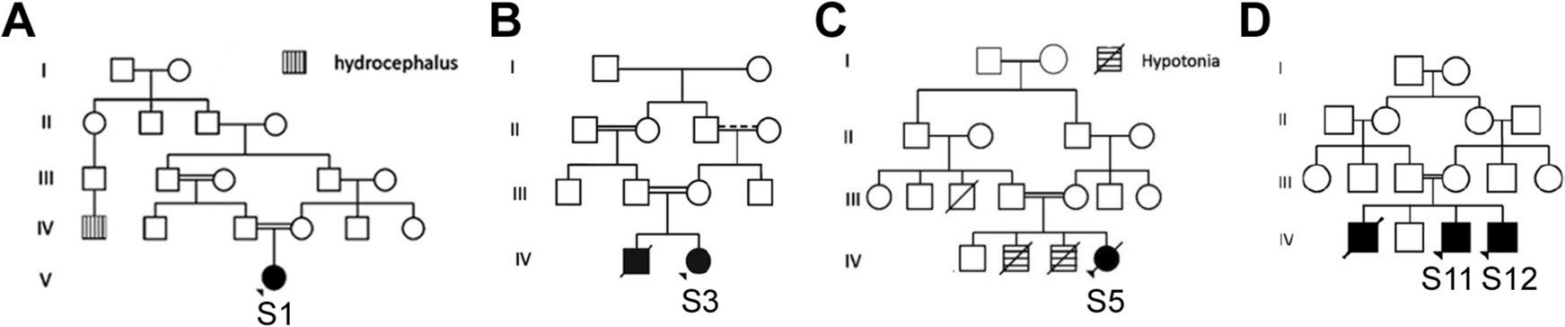
Pedigrees for cases with family history of CMD. **A** S1 was born from a 1^st^ cousin union and had a distant cousin diagnosed with hydrocephalus. **B** S3 was born from a 1^st^ cousin union and had a deceased younger brother with hydrocephalus and failure to thrive. **C** S5 was born from a 1^st^ cousin union and had two siblings with severe hypotonia who died in infancy. **D** S11 and S12 are siblings and had an older sib with CMD who died at age 9

All of the cases in Group I and II were born from consanguineous unions, apart from S2. However, both parents of S2 originated from the same village. The age at the time of the first visit to the genetic clinic was significantly younger in Group I (1 day to 7 months old) than Group II (2 to 7 years old). Several cases had a family history suggestive of CMDs. For instance, S1 had a distant cousin with hydrocephalus (Fig. 1A), S3 had one deceased sib with hydrocephalus and failure to thrive (Fig. 1B), S5 had two siblings who died during the neonatal period suffering from severe hypotonia (Fig. 1C), and S7 had a distant relative with late onset muscle disease. Among cases of Group II, S11 and S12 are siblings with a previous history of an older sibling with the same condition who died at age of 9 (Fig. 1D).

Except for S7, individuals in Group I exhibited severe global developmental delay with no motor development present in S1, S2, and S6 (Table 1). They had no head control and failed to reach any motor milestones. Most individuals had highly elevated CK levels and myopathic changes were seen on EMG. No muscle biopsies were conducted. Additionally, each individual in Group I had absent speech, eye abnormalities ranging from ptosis to microphthalmia and retinal detachment, and five of them presented with seizures that were controlled by anti-epileptic drugs (AEDs). Four patients (S3-6) died before the age of two. Interestingly, S7 presented with milder clinical manifestations compared to the rest of Group I. During the last follow-up at 30 months of age, he showed typical age-appropriate behaviors, including responding to his name and following simple instructions, but he was unable to speak. The maximum motor achievement was sitting without support by age 1. He had highly elevated CK and only mild ptosis in his left eye.

Individuals in Group II achieved further milestones than individuals in Group I. Although none had achieved independent ambulation, four out of the five individuals (S8, S9, S11, S12) were able to sit without support by the age of two. S8 learned to write despite the presence of contractures and S10 only had neck support. Contractures were present in 3 patients (S8, S10, S11) and were progressive, affecting mainly knees and elbows. CK levels were not as elevated compared to Group I, but all showed myopathic changes upon EMG analysis. In addition, all individuals in Group II had normal cognitive function and behavior and none had seizures or ocular abnormalities. S8 and S10 had normal language development, while S9 only started to speak at 3 years of age and the siblings S11 and S12 said the first words at 2 years of age and had a mild deficit in articulation despite an IQ result in the typical range.

### Radiological analysis of brain abnormalities

**Fig. 2:**
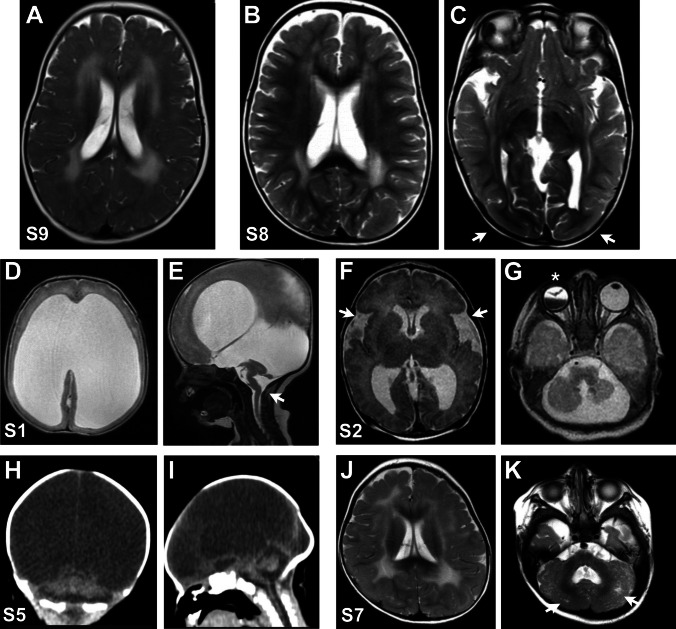
Brain imaging for selected cases. **A** Axial T2 MRI image showing the classical T2 hyperintense signal within the periventricular white matter in case S9. **B**, **C** Axial T2 MRI images of case S8 showing abnormal hyperintense signal within the periventricular white matter with additional cortical malformations including bilateral occipital segmental lissencephaly (arrows in **C**). **D**, **E**: Axial (**D**) and sagittal (**E**) T2 MRI images of case S1 showing diffuse cobblestone type II lissencephaly with flattening of the entire cerebral mantle as well as hypoplastic cerebellum and pons and Z-shaped brainstem (arrow in **E**). **F**, **G**: Different axial T2 MRI images of case S2 showing supratentorial abnormal perisylvian cortical development (arrows in **F**), cerebellar hypoplasia, small size pons, and abnormal right eye globe with abnormal right lens configuration and intra vitreous hemorrhage (asterisk in **G**). **H**, **I**: Coronal (**H**) and sagittal (**I**) non contrast CT images of case S5 showing the supratentorial brain nearly replaced by CSF apart from the interhemispheric fissure, also associated with hypoplastic cerebellum and kinked brain stem structures. **J**,** K**: Axial T2 MRI images of case S7 showing periventricular white matter abnormalities in addition to hypoplasia of both cerebellar hemispheres with multiple bilateral microcysts as well as dysplastic features

The findings of the brain MRI further supported the suspected diagnoses. All individuals in Group II showed the classical abnormal white matter myelination manifested as symmetrical T2 hyperintense signal in the periventricular and deep white matter that is typically observed in merosin-deficient CMD (Fig. 2A). Brain MRI for S8 also showed additional cortical malformations including bilateral occipital lissencephaly (Fig. 2B–C).

In contrast, Group I had a more variable presentation in the WWS/MEB spectrum. Brain MRI for S1, S4, and S6 showed lissencephaly with cobblestone malformation with flattening of the entire cerebral mantle as well as hypoplastic cerebellar hemispheres, cerebellar vermis, and z-shaped hypoplastic pons (Fig. 2D–E). S2 had supratentorial ventricular dilatation, abnormal perisylvian cortical development, cerebellar hypoplasia, microcysts, small size pons, and hypertrophied tectal plates (Fig. 2F–G). Only non-contrast Computed Tomography (CT) imaging was available for S5 showing the supratentorial brain nearly replaced by CSF apart from the interhemispheric fissure, and hypoplastic cerebellum and brain stem structures (Fig. 2H–I). S7 had white matter abnormalities in addition to hypoplasia of both cerebellar hemispheres with multiple bilateral microcysts as well as dysplastic features (Fig. 2J–K).

### Whole exome sequencing analysis

DNA from all affected individuals and their parents was sent for WES. Initial analysis of known CMD genes reached a genetic diagnosis in 71% (5/7) of Group I with two homozygous variants in *FKRP* and one homozygous variant each in *POMT1*, *POMK*, and *B3GALNT2*. In Group II, a diagnosis was reached for 100% (5/5) of cases, as all had homozygous truncating variants in *LAMA2* (Table 2). All parents were confirmed to be heterozygous carriers apart from S6 where parental DNA was not available.

**Table 2:** Variants identified in this study

CASE	GENE	REFSEQ ACCESSION	DNA	PROTEIN	CADD SCORE	ACMG SCORE	REFERENCE
S1	*POMK*	NM_032237.5	c.641A > T	p.Gln214Leu	24.6	VUS	Not reported
S2	*POMT1*	NM_007171.4	c.2179_2180del	p.Ser727fs		Pathogenic	27
S3	*FKRP*	NM_024301.5	c.778G > T	p.Glu260Ter		Pathogenic	Not reported
S4	*B3GALNT2*	NM_152490.5	c.1338G > A	p.Trp446Ter		Pathogenic	Not reported
S5	*FLVCR1*	NM_014053	c.215dupC	p.E74Rfs			Not reported
S7	*FKRP*	NM_024301.5	c.1364C > A	p.Ala455Asp	29.8	Pathogenic	28, 29, 30
S8	*LAMA2*	NM_000426.4	c.6955C > T	p.Arg2319Ter		Pathogenic	31, 32
S9	*LAMA2*	NM_000426.4	c.4645C > T	p.Arg1549Ter		Pathogenic	33
S10	*LAMA2*	NM_000426.4	c.3886_3889del	p.Ile1296AlafsTer		Pathogenic	Not reported
S11-12	*LAMA2*	NM_000426.4	c.6636_6637del	p.Gly2213SerTer		Pathogenic	Not reported

S1 had a homozygous missense variant in *POMK* (NM_032237.5:c.641A > T; NP_115613.1:p.Gln214Leu). This variant is not present in population databases (gnomAD, GME), never reported in literature, and highly conserved among different species. Algorithms developed to predict the effect of missense changes on protein structure and function (SIFT, PolyPhen-2, CADD) suggest that this variant is likely to be disruptive. S2 had 2 bp frameshift deletion in *POMT1* (NM_007171.4:c.2179_2180del; NP_009102.4:p.Ser727fs) which creates a premature stop codon and has been previously reported in two other cases [28]. S3 had a stop-gain variant in *FKRP* (NM_024301.5:c.778G > T; NP_077277.1:p.Glu260Ter) which is absent in gnomAD and GME. This variant has been only reported 3 times in ClinVar. S4 had a stop-gain variant in *B3GALNT2* (NM_152490.5:c.1338G > A; NP_689703.1:pTrp446Ter) which is not found in gnomAD, GME, but is present in one individual in ClinVar. S7 had a missense variant in *FKRP* (NM_024301.5:c.1364C > A; NP_077277.1:p.Ala455Asp) that has been previously reported in the Tunisian, Moroccan, and Arab populations [29–31].

In the merosin-deficient CMD group, WES identified 4 different homozygous variants in *LAMA2*. S8 had a homozygous stop-gain variant (NM_000426.4:c.6955C > T; NP_000417.3:p.Arg2319Ter) that has been reported twice in trans with other *LAMA2* variants [32, 33]. S9 had a homozygous stop-gain *LAMA2* (NM_000426.4:c.4645C > T; NP_000417.3:p.Arg1549Ter) which has been reported in several studies [34–36]. S10 had a novel 4 bp deletion in *LAMA2* (NM_000426.4:c.3886_3889del; NP_000417.3:p.Ile1296AlafsTer), and the siblings S11 and S12 had a novel 2 bp frameshift deletion in *LAMA2* that lead to premature stop codon (NM_000426.4:c.6636_6637del**;** NP_000417.3:pGly2213SerTer).

Following WES analysis of protein-altering variants, cryptic splicing analysis, and CNV prediction, only two cases, S5 and S6, remained genetically unsolved. In S5, five candidate genes were left following rare variants filtering (*PIP5K1C, PRX, HRC, ADAT3,* and *FLVCR1*). Among these, the only protein-truncating variant was a novel homozygous frameshift insertion in *Feline Leukemia Virus subgroup C Receptor 1* (*FLVCR1*; NM_014053:c.215dupC; NP_054772.1:p.E74Rfs). In S6, coverage-based CNV prediction with ExomeDepth predicted a CNV encompassing *POMK* in addition to several of its inferred enhancers listed in the GeneHancer database [37]. However, we are unable to confirm whether this predicted duplication is de novo or inherited, and further experiments would be required to confirm its presence and determine its effect on *POMK* expression. We also identified rare, homozygous variants in four possible candidate genes (*DCTN2*, *DNAH5*, *GBE1*, and *NEURL1)*. Of interest was a homozygous missense variant in *NEURL1* (NM_004210.5:c.1051G > T; NP_004201.3:p.Gly351Cys). This variant was absent in gnomAD and GME, occurred at a conserved amino acid, and is predicted to be damaging by SIFT (0.002), PolyPhen2 [1], and CADD [27]. Overall, we identified variants in 86% (6/7) of individuals in Group I and 100% (5/5) of individuals in Group II, with candidate variants in S6 where further investigation is warranted.

## Discussion

In this study, we present one of the first cohorts from Egypt comprised of patients with CMD and brain malformations. Each patient underwent a comprehensive clinical workup and were grouped as those with suspected dystroglycanopathy (Group I) or suspected merosin-deficient CMD (Group II). However, this workup is not always sufficient to provide a definitive diagnosis, especially given the clinical heterogeneity of brain malformations observed in severe CMDs. As such, we leveraged WES to identify variants causing dystroglycanopathies and merosin-deficient CMD, several of which were novel or reported for the first time in Egypt. Overall, WES represents a powerful technology for detecting disease causing variants.

The diagnostic rate achieved in this study was very high with a potentially disease-causing mutation detected in 86% (6/7) of individuals in Group I (suspected dystroglycanopathy) and 100% (5/5) in Group II (suspected merosin-deficient CMD). All variants identified in this study were found in homozygosity despite only 9/12 enrolled probands being born from first cousin (consanguineous) parents. It is estimated that about 30% of all marriages in Egypt in the last 40 years have been consanguineous [38]. Hence, our data emphasizes the higher burden of severe, rare diseases in consanguineous populations since referral to our center or recruitment in this study was not restricted to consanguineous unions. The lack of a national registry system for rare diseases along with the insufficiency of diagnostic tools makes it difficult to reach the precise rate of rare disorders in Egypt. However, a large study conducted at one genetic center at Cairo University in the period between 1966 to 2009 showed that the frequency of genetic disorders was 4.3% [39]. In addition, about 23,000 families with known or suspected genetic disorders were registered in the genetics clinics at the Medical Research Institute since 1981, and several studies of specific genetic disorders were conducted to underline not only the frequency but also the genetic makeup of these syndromes in Egypt [40, 41].

One of the goals of our study was to define whether common founder mutations in specific genes for dystroglycanopathies were present in the Egyptian population. The number of studies that have focused exclusively on this type of CMD is limited with diverse population-specific variants being identified. For example, a Middle Eastern founder mutation in *LAMA2* (c.3924 + 2 T > C) that causes an in-frame deletion of 63 amino acids was identified in four families from Saudi Arabia and one family from Sudan [42, 43]. This variant likely originated in Saudi Arabia and later entered North Africa, which may be relevant to individuals with merosin-deficient CMD in Egypt [42, 44]. Furthermore, a founder *FKTN* variant accounts for the majority of severe CMD (FCMD) in Japan, China, and South Korea [10, 11, 45], and a different founder mutation in *FKTN* leads to WWS in Ashkenazi Jews [8, 12]. In contrast, *POMT1* and *POMGNT1* account for 30–40% of MEB/WWS cases in European populations [8]. Among the dystroglycanopathy cohort, we detected pathogenic variants in 4 different genes in 5 cases. The only gene that was found mutated twice was *FKRP* with two different variants causing variable clinical manifestations: S3 harbored a novel stop-gain variant and had WWS with cerebellar microcysts and hydrocephalus, while S7 harbored a missense variant and presented with a phenotype overlapping MEB and merosin-deficient CMD characterized by white matter hyperintensities, cerebellar hypoplasia, and microcysts without hydrocephalus or cortical malformation.

The missense variant in *FKRP* identified in S7 (p.Ala455Asp) was previously reported as a founder mutation in the Tunisian population and was also reported in other families of Arabian descent [29–31]. This is the only Arab/North African founder mutation identified in our cohort. Further, this variant is associated with a variable clinical presentation [30]. Our case differs from previous cases with severe developmental delay in having appropriate behavioral responses for his age despite the lack of language. This could suggest the presence of unidentified modifiers of *FKRP.*

The remaining three genetically confirmed dystroglycanopathy cases each had variants in different genes. While variants in *POMT1* are the most frequent cause of WWS in other populations, we only found one previously reported *POMT1* frameshift variant in a single patient with a presentation consistent with MEB (S2). This variant also leads to variable presentation with two previous cases diagnosed with WWS and MEB [28]. Both presented with microcephaly like S2, but also had contractures which were absent in S2. The brain MRI abnormalities in the previous MEB case were similar to S2: hydrocephalus, cerebral hypoplasia with cerebral microcysts, in addition to brain stem abnormalities. In one of the WWS cases (S1), we identified a missense variant in *POMK*, which is a very rare cause of dystroglycanopathy. To date, only nine pathogenic *POMK* variants have been reported worldwide in 16 cases, and five of them were stillbirth or fetuses from induced termination of pregnancy [46]. Six out of nine variants cause WWS while the remaining three cause limb-girdle muscular dystrophy [46]. *POMK* genotype/phenotype correlations are complex because even variants leading to expression of a significantly truncated protein can result in a mild phenotype [47]. However, the functional and physiological mechanisms underlying the phenotypic variability remain unclear. We also identified a novel homozygous nonsense variant disrupting the glycosyltransferase domain of *B3GALNT2* leading to WWS. *B3GALNT2* variants also lead to variable presentations with 23 cases documented in the literature: 5 having WWS, 10 having MEB, and 8 with atypical CMD [48].

In S5, we identified a likely pathogenic variant in *FLVCR1*, which is not a known cause of dystroglycanopathy. FLVCR1 was shown to act as a heme transporter and was recently proposed to be a major choline transporter [49]. It is widely expressed but is most abundant in the retina, spinal cord, and brain, especially in the cerebellum and hippocampus [50]. Missense variants in *FLVCR1* cause posterior column ataxia with retinitis pigmentosa [50], and homozygous protein-truncating variants have never been reported, though removal of *Flvcr1* in mice is embryonic lethal [49]. However, loss of function variants of its paralog *FLVCR2* cause a syndrome characterized by proliferative vasculopathy and hydranencephaly-hydrocephaly, also termed Fowler Syndrome [51]. Eye abnormalities, joint contractures, and muscle atrophy are also common in Fowler Syndrome. S5 presented with profound hydrocephaly, contractures, seizures, and optic nerve atrophy which would also be consistent with this syndrome. Since only a CT was available for this case, only severe hydrocephalus was ascertained leading to the possible dystroglycanopathy diagnosis. In addition, there is no autopsy information to confirm the distinctive vasculopathy. Considering the clinical overlap, it is likely that homozygous loss of function variants in *FLVCR1* also cause a presentation consistent with Fowler Syndrome.

S6 was the only case in Group I that remained unsolved after a comprehensive analysis of rare variants. Among the variants identified, one potential candidate is *NEURL1*, which encodes an E3 ubiquitin ligase that plays a role in the regulation of Notch pathway and is expressed at variable levels throughout the muscle and brain [52]. Multiple regulators of the Notch pathway have been involved in muscle disease (*MEGF10*, *POGLUT1*, and *JAG2*) [53]. Furthermore, a unique variant in *JAG1*, which is not directly related to skeletal muscle disease, has been found to possess a modifying effect on muscular dystrophy [54], and *NEURL1* had been found to affect the signaling activity of *JAG1* by directly enhancing its ubiquitination [55]. Further investigations may be warranted to determine whether *NEURL1* mutations could lead to dystroglycanopathy phenotypes. Additionally, we used ExomeDepth to predict CNVs from WES coverage data, as pathogenic CNVs have been identified in individuals with dystroglycanopathy in several studies including a ~ 63 kb intragenic deletion in *LARGE1* in a case with WWS [56] and a ~ 1.6 kb deletion in *POMGNT1* in an individual with MEB [57]. This strategy indicated that S6 may harbor a heterozygous duplication encompassing *POMK* and several of its inferred enhancers. While experimental assays have shown that ExomeDepth CNV predictions are often accurate [58], further analysis is warranted to confirm the presence of this CNV and evaluate its effect on *POMK* expression.

In Group II (suspected merosinopathy), we identified 4 different homozygous truncating variants in *LAMA2*: two stop-gain, and two frameshift deletions. The two frameshift deletions were novel, and the stop-gain variants p.Arg2319Ter and p.Arg1549Ter were reported eight and 14 times in the LOVD database, respectively (https://databases.lovd.nl/shared/genes/LAMA2). The p.Arg2319Ter mutation was associated with bilateral occipital pachygyria in addition to classical white matter changes, but this type of cortical malformation is not uncommon for individuals with merosin deficiency [4, 39]. In addition to S8, this variant was reported twice in trans with other variants: once with a deletion of exon 56 (c.7750_7899del) in a patient with hypotonia and severe dystrophic changes in muscle biopsy [32], and once with p.Cys2909Arg in a patient with late onset merosin-deficient CMD [33]. The variant identified in S9 (p.Arg1549Ter) has been reported several times in other studies with profound clinical variability. One patient with complete merosin deficiency was 17 years old and sitting without support, which was his maximum motor achievement [34]. Another patient presented with only partial merosin deficiency. They achieved ambulation with aid and were able to climb stairs with support [35]. Lastly, this mutation was detected in a compound heterozygosity in a patient with CMD, dilated cardiomyopathy, and life-threatening ventricular arrhythmias [36]. Overall, none of the patients in Group II could achieve independent walking, consistent with the findings that all the patients who carried biallelic premature termination codons never walk [3].

As the rate of genetic diagnosis for merosin-deficient CMD in our study is 100%, which has also been achieved in another study [59], we recommend that following clinical and radiological assessment, *LAMA2* gene sequencing should be performed first in similar cases. However, availability and cost effectiveness remain two major concerns. For the dystroglycanopathies, we found mutations in 86% of cases in this study, but future studies on larger cohorts may benefit from additional NGS assays such as whole genome sequencing with long-read strategies, as we suspect noncoding variants that go undetected in WES may contribute to many unsolved cases.

## Data Availability

Data reported in this study will be made available upon request from the corresponding author.
